# Cholera Epidemiology in Nigeria: an overview

**Published:** 2012-07-02

**Authors:** Ajoke Olutola Adagbada, Solayide Abosede Adesida, Francisca Obiageri Nwaokorie, Mary-Theresa Niemogha, Akitoye Olusegun Coker

**Affiliations:** 1 Molecular Biology and Biotechnology Division, Nigerian Institute of Medical Research, Yaba, Lagos, Nigeria; 2 Department of Medical Microbiology and Parasitology, College of Medicine, University of Lagos, Lagos, Nigeria

**Keywords:** Cholera, Nigeria, epidemiology, Vibrio cholerae

## Abstract

Cholera is an acute diarrhoeal infection caused by ingestion of food or water contaminated with the bacterium, *Vibrio cholera*. Choleragenic *V. cholera* O1 and O139 are the only causative agents of the disease. The two most distinguishing epidemiologic features of the disease are its tendency to appear in explosive outbreaks and its predisposition to causing pandemics that may progressively affect many countries and spread into continents. Despite efforts to control cholera, the disease continues to occur as a major public health problem in many developing countries. Numerous studies over more than a century have made advances in the understanding of the disease and ways of treating patients, but the mechanism of emergence of new epidemic strains, and the ecosystem supporting regular epidemics, remain challenging to epidemiologists. In Nigeria, since the first appearance of epidemic cholera in 1972, intermittent outbreaks have been occurring. The later part of 2010 was marked with severe outbreak which started from the northern part of Nigeria, spreading to the other parts and involving approximately 3,000 cases and 781 deaths. Sporadic cases have also been reported. Although epidemiologic surveillance constitutes an important component of the public health response, publicly available surveillance data from Nigeria have been relatively limited to date. Based on existing relevant scientific literature on features of cholera, this paper presents a synopsis of cholera epidemiology emphasising the situation in Nigeria.

## Background

Cholera caused by *Vibrio cholera* continues to be a global threat to public health and a key indicator of lack of social development. Once common throughout the world, the infection is now largely confined to developing countries in the tropics and subtropics. It is endemic in Africa, parts of Asia, the Middle East, and South and Central America. In endemic areas, outbreaks usually occur when war or civil unrest disrupts public sanitation services. Natural disasters like earthquake, tsunami, volcanic eruptions, landslides and floods also contribute to outbreak by disrupting the normal balance of nature [1]. This creates many health problems, food and water supplies can become contaminated by parasites and bacteria when essential systems like those for water and sewage are destroyed. Developing countries are disproportionately affected because of their lack of resources, infrastructure and disaster preparedness systems [2, 3]. In newly affected areas, outbreaks may occur during any season and affect all ages equally. The organism normally lives in aquatic environments along the coast. People acquire its infection by consuming contaminated water, seafood, or other foods. Once infected, they excrete the bacteria in stool. Thus, the infection can spread rapidly, particularly in areas where human waste is untreated.

In Nigeria, the infection is endemic and outbreaks are not unusual. In the last quarter of 2009, it was speculated that more than 260 people died of cholera in four Northern states with over 96 people in Maidugari, Biu, Gwoza, Dikwa and Jere council areas of Bauchi state [4]. Most of the Northern states of Nigeria rely on hand dug wells and contaminated ponds as source of drinking water. Usually, the source of the contamination is other cholera patients when their untreated diarrhoea discharge is allowed to get into water supplies [4].

**Figure 1:**
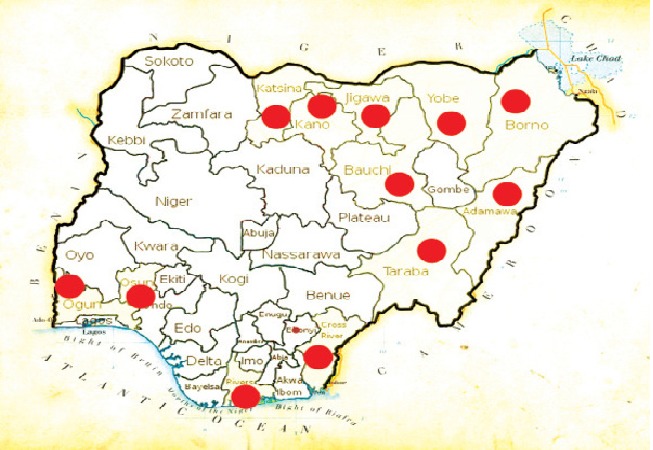
Main regions affected by 2010 cholera outbreak in Nigeria. Source: http://reliefweb.int/sites/reliefweb.int/files/resources/29A1BC92996A920385257792005A76BE-Full_Report.pdf

The 2010 outbreak of cholera and gastroenteritis and the attendant deaths in some regions in Nigeria brought to the forefront the vulnerability of poor communities and most especially children to the infection. The outbreak was attributed to rain which washed sewage into open wells and ponds, where people obtain water for drinking and household needs. The regions ravaged by the scourge include Jigawa, Bauchi, Gombe, Yobe, Borno, Adamawa, Taraba, FCT, Cross River, Kaduna, Osun and Rivers. Figure 1 depicts major outbreak locations. Even though the epidemic was recorded in these areas, epidemiological evidence indicated that the entire country was at risk, with the postulation that the outbreak was due to hyper-virulent strains of the organism [5].

In Nigeria, the first series of cholera outbreak was reported between 1970- 1990 [6]. Despite this long experience with cholera, an understanding of the epidemiology of the disease aiding its persistence in outbreak situations is still lacking. This review therefore provides the knowledge gaps of the infection with the hope that it will help to develop targeted approaches to controlling the infection.

### Description and Characteristics of *Vibrio cholera*

*Vibrio cholera*, a curved Gram-negative bacillus belongs to the family, Vibrionaceae and shares some characteristics with the family, Enterobacteriaceae [7]. The species *V. cholera* comprises both pathogenic and nonpathogenic strains. *Vibrio cholera* O1 and O139 are the only serotypes responsible for the disease defined clinically and epidemiologically as cholera [8, 9]. *Vibrio cholera* O1 is divided into classical and El Tor biotypes, and into three serosubtypes-Ogawa, Inaba, and Hikojima. *Vibrio cholera* O139 has characteristics in common with the El Tor biotype but differs from O1 in its polysaccharide surface antigen [10]. Cholera cases are confirmed through the isolation of *Vibrio cholera* O1 or O139 from stools in any patient with diarrhea [11]. Other serovars of *V. cholera* are generally termed non-O1, non-O139 strains. They are non-choleragenic, usually cause a milder form of gastroenteritis than O1 and O139, and are normally associated with sporadic cases and small outbreaks rather than with epidemics and pandemics [12]. *Vibrio cholerae* 01 Eltor is the commonest strain in Nigeria [13–16].

About 75% of people infected with *V. cholera* do not develop any symptoms, although the bacteria are present in their faeces for 7-14 days after infection and are shed back into the environment, potentially infecting other people. Among people who develop symptoms, 80% have mild or moderate symptoms, while around 20% develop acute watery diarrhoea with severe dehydration [17]. In severe infections, more than one quart of water and salts is lost per hour. The stool looks gray and has flecks of mucus in it- termed “rice water stools”. Within hours, dehydration can become severe, causing intense thirst, muscle cramps, and weakness. Very little urine is produced and the eyes may become sunken, and the skin on the fingers may become much wrinkled. If dehydration is not treated, loss of water and salts can lead to kidney failure, shock, coma, and death. In people who survive, symptoms usually subside in 3 to 6 days. Most people are free of the bacteria in two weeks. The bacteria remain in a few people indefinitely without causing symptoms.

The most important virulence factor associated with *V. cholera* O1 and O139 is the cholera toxin (ctx). The ctx genes (ctxA and ctxB) encoding the production of the cholera toxin have been sequenced and these have enabled development of deoxyribonucleic acid (DNA) probes and polymerase chain reaction (PCR) methods for detection of the organism [18–23]. In addition to cholera toxin, choleragenic strains of *V. cholera* possess the ability to adhere to, and colonise, the small intestine (colonisation factor), which has been ascribed to a toxin co-regulated pilus (TCP). Genes encoding major virulence-associated factors are found in clusters [24]. It has been shown that ctx genes form part of a filamentous bacteriophage designated CTX phage [25, 26]. The pilus colonisation factor is also known to act as a receptor for the CTX phage [27, 28] and is encoded by the tcpA gene that is part of the *V. cholera* pathogenicity island [29, 30].

A complex cascade of regulatory proteins that control expression of *V. cholera* virulence determinants has been reported. For instance, in responding to the chemical environment at the intestinal wall, the organism produces the TcpP/TcpH proteins, which, together with the ToxR/ToxS proteins, activate the expression of the ToxT regulatory protein. ToxT then directly activates expression of virulence genes that produce the toxins, causing diarrhea in the infected person and allowing the bacteria to colonise the intestine [31, 32]. Although reports on characteristics of Nigerian strains are insufficient [13] implied that *Vibrio cholerae* from bi-weekly surveillance cultures for a period of eight months were concordant with strains analysed at the onset of their study. It may therefore be assumed that pathogenic potential and characteristics of *V.cholerae* in Nigeria are similar to those that have been studied elsewhere.

### History and geographical distribution

Historical data shows that there have been eight pandemics of cholera worldwide, of which the seventh and eighth continue (Table 1). During the 19th century, cholera spread across the world from its original reservoir in Bangladesh and West Bengal [29], claiming millions of lives across all continents most especially Africa and Asia. The cholera outbreak in Bangladesh in 2004 was post flooding involving more than 17 000 cases, with the isolation of *Vibrio cholera* (O1 Ogawa and O1 Inaba) [1]. The seventh pandemic started in South Asia in 1961, reached Africa in 1971 and the Americas in 1991 [33, 34]. Cholera is now endemic in many countries and recent studies have indicated that global warming creates a favourable environment for the bacteria [35].

**Table 1:** Cholera pandemics, 1817 till present

Pandemic	Years	Origin	Other regions affected	Pathogen
First	1817-1823	India	South-east Asia, Middle East and East Africa	*Vibrio cholerae* serogroup O1 biotype classical
Second	1829-1851	India	South-east Asia, Middle East Europe, America and Africa	*Vibrio cholerae* serogroup O1 biotype classical
Third	1852-1859	India	South-east Asia, Middle East Europe, America and Africa	*Vibrio cholerae* serogroup O1 biotype classical
Fourth	1863-1879	India	South-east Asia, Middle East Europe, America and Africa	*Vibrio cholerae* serogroup O1 biotype classical
Fifth	1881-1896	India	South-east Asia, Middle East Europe, America and Africa	*V. cholerae* O1, classic
Sixth	1899-1923	India	South-east Asia, Middle East Europe, America and Africa	*V. cholerae* O1, classic
Seventh	1961 to present	Sulawesi (Celebes), Indonesia	South-east Asia, Middle East Europe, South America and Africa	*V. cholerae* O1, El Tor
Eighth	1992 to present	Madras, India	South-east Asia	*V. cholerae* O139

Adapted from [34, 59]

Although reports of cholera epidemic in Nigeria have not been consistent, the disease is very dynamic. The emergence of cholera was evident in 1970 and was re-introduced in 1991. During the last two decades, three major epidemics have occurred: 1992 [36], 1995-1996 [37], and 1997 [15, 37]. The northern Nigeria has been known to be endemic for cholera infection. Epidemiological data from Public Health Department of Kano State Ministry of Health, Northern Nigeria, revealed that the frequency and distribution of recurrent cholera epidemics in the state during 1995 to 2001, were 2 630 in 1995/1996, 847 in 1997 and 2, 347 in 1999 [15]. In Jos, North Central Nigeria, [14] observed that all isolated strains were *Vibrio cholerae* 01 Eltor of Inaba serotype. The authors concluded that *Vibrio cholerae* 01 is endemic in Jos, Nigeria.

Analysing genetic variation in isolates of *V. cholera* O1 and O139 from successive outbreaks of cholera and the determination of whether these genetic variations contribute to the emergence of new clones of *V. cholera* can be an important step in understanding the evolution of new pathogenic strains. Molecular analysis of the pandemic isolates of *V. cholera* showed limited genetic grouping of pandemic isolates. So far, two clusters have been identified by Amplified fragment length polymorphism (AFLP): Cluster I and Cluster II. Based on the changes in the clonal structure, Cluster I basically consist of strains from the 1960s and 1970s, while Cluster II contains strains from the 1980s and 1990s and revealed a temporal pattern of change in the clone. This is particularly apparent in the strains from the African Continent [38]. Although, it seems that the Northern part of Nigeria bears much of the burden of cholera, very little is known about the characteristics of circulating strains.

## Infection pattern and seasonality

Cholera infection rate, sex and age distribution and seasonality are not constant. In 1982, Katsina, Nigeria, was affected by an outbreak of gastroenteritis associated with *Vibrio cholera* serotype ′Ogawa′ [36]. The overall case fatality rate was 7.7%. During the Calabar, south southern part of Nigeria outbreak, adults and those in the 11-20 and 21-30 age groups accounted for most of the cases regardless of sex [39]. The report from Jos (North-central) indicated that age group 20-29 years had the highest isolation rate [14]. The 1996 outbreak reported in Kano, Northern Nigeria affected 1, 384 individuals with a fatality rate of 5.3% [37]. Children were the most affected among all age groups and accounted for 22% of the total cases reported in Ibadan, southwest Nigeria [6]. The wave of the *El Tor* cholera pandemic that occurred in 1991 had a case fatality ratio of 13% in Nigeria [40].

In Abeokuta, South-western Nigeria, between November 2005 and January 2006, 11 deaths from the 115 cases with case fatality rate of 9.6% were reported from a cholera outbreak [16]. The 2010 outbreak was projected as the worst in Nigeria since 1991 with the highest case-fatality rates [41]. The Nigerian states with case fatality rates (CFRs) in the 2010 outbreak include Plateau, Kaduna and Katsina states at 23.0%, 9.0% and 7.6% respectively. Women and children accounted for 80% of reported cases [41]. All outbreak studies carried out in Nigeria till 1999 are summarised on Table 2. Table 3 shows cholera incidence and case fatality rate by state. Despite this, the occurrence of the hyper-infectivity of the organism remains largely unknown and no documented epidemiological information on the infecting strains or patients. We believe that studies are required to understand its prevalence and nature of infection in the country.

**Table 2:** Data from studies on major cholera outbreaks in Nigeria, 1991-1999

Year	State	Source of Infection	Number of reported cholera cases	Case fatality rates	Serotypes	Age group	References
1991	Cross River		588	13%	*V. cholerae* 01 (biotype El Tor, serotype Ogawa)	Not indicated	[39]
1992	Katsina	Well water	662	7.7%	Ogawa	11-30years	[36]
1995/6	Kano	Water sources	2,630	15%	El-Tor	0-5 years	[15, 37]
1997			847	5%			
1999			2,347	2%			
1996	Ibadan		1,384	5.3%		Children (age range, not indicated	[6]

**Table 3:** Cholera incidence and case fatality rate during 2010 outbreak

	State	Geographical Zone	LGAs in state	LGAs affected	Total cases	Total deaths	Case fatality rate
1	Rivers	South south	19	9	314	13	4.1
2	Cross River	South south	15	4	319	8	2.5
3	Osun	South west	30	1	87	2	2.3
4	Ekiti	South west	16	2	381	0	-
5	Adamawa	North east	21	12	1,816	104	5.7
6	Bauchi	North east	20	20	7,783	175	2.2
7	Borno	North east	27	21	5,822	264	4.5
9	Gombe	North east	11	11	1,998	113	5.7
10	Jigawa	North west	27	13	632	35	5.5
11	Kano	North west	44	11	513	27	5.3
12	Plateau	North central	17	5	526	29	23.0
13	Taraba	North east	17	5	502	35	7.0
14	Yobe	North east	17	12	2,009	129	6.4
15	Zamfara	North west	14	5	1,458	58	4.0
16	Kaduna	North west	23	7	301	27	9.7
17	Katsina	North west	19	18	2,128	162	7.6
18	Sokoto	North west	23	1	51	1	2.0
	**Total**		**360**	**157**	**26,240**	**1,182**	**4.5**

Source: [42]. LGA = Local Government Authority

Nevertheless, cholera exists as a seasonal disease, occurring mostly during rainy seasons. Pascual and colleagues highlighted the importance of rainfall as a driver of the seasonal cycle of cholera through its waterborne transmission, its dose-dependent nature of infection, and the decline of cases during the rainy season [42]. Higher number of cases reported in Kano, Nigeria occurred during the rainy season [36]. In Calabar, South-southern part of the country, the incidence of cholera mostly occurred during the dry season followed by subsidence at the onset of rainy season [39]. Consequently, seasonality of infection is not a critical issue in Nigeria as infections have been reported in both rainy and dry seasons.

## Risk factors for increased transmission

For a cholera outbreak to occur, two conditions have to be met: there must be significant breaches in the water, sanitation, and hygiene infrastructure used by groups of people, permitting large-scale exposure to food or water contaminated with *Vibrio cholera* organisms; and cholera must be present in the population. Cholera has been proven to be transmitted through fecal-oral route via contaminated food, carriers of the infection and inadequate sanitary conditions of the environment. The principal mode of transmission however remains ingestion of contaminated water or food.

In Nigeria, the 1996 cholera outbreak in Ibadan (Southwest) was attributed to contaminated potable water sources [6]. Street vended water and not washing of hands with soap before eating food are possible reasons for the 1995-1996 cholera outbreaks in Kano state [35]. Drinking water sold by water vendors was also connected with increased risk of contracting the disease. In Katsina, the outbreak of the disease was linked to faecal contamination of well water from sellers [36]. The recent 2010 outbreak of cholera was speculated to be directly related with sanitation and water supply. The hand dug wells and contaminated ponds being relied on by most of the Northern states as source of drinking water was a major transmission route during the outbreak. Perhaps, these wells were shallow; uncovered and diarrhoea discharge from cholera patients could easily contaminate water supplies [4].

Another factor that may greatly contribute to risk of cholera transmission is population movement which enhances the spread of the infectious agent to others and to different sites. For instance, all the surviving residents that fled a two month outbreak in Kebbi state (North-north) became indices for subsequent infection in the north and southern part of a neighbouring state [43]. Additional overcrowding increases risk of contact with vomitus, excreta and contaminated water or food. Since early detection and containment of cases (isolation facilities) are paramount in reducing transmission, poor access to health services and poor diagnosis may become major barrier to controlling the infection. Lack of safe water and poor sanitation are important risk factors. All these features have contributed greatly to cholera infections in Nigeria.

### Host Genetic Factors, Demographic and Socioeconomic Factors

Susceptibility to cholera infection and factors enhancing its spread is multi-factorial. The host immune system is the critical defence mechanism against cholera. However, infection with cholera can result in a range of responses, from severe and life threatening diarrhoea to mild or unapparent infections. Another factor is differences in gastric acidity. It been stated that low acid production can lead to increased susceptibility to cholera [44]. People who produce less stomach acid such as young children, older people, and those taking drugs that reduce stomach acid, including proton pump inhibitors (such as omeprazole) and histamine-2 (H2) blockers (such as ranitidine) [45] are likely to contact the infection.

A number of demographic and socioeconomic factors including age, gender, nutritional status, social status, economic status and travel abroad are also known to play crucial role in susceptibility to choleragenic *V. cholera*. Sanitation and nutrition are particularly important factors and it has become clear that good sanitation and hygienic practices largely prevent the disease. *Vibrio cholera* infection is known to be more severe in individuals suffering from malnutrition. Hypochlorhydria associated with malnutrition, B12 deficiency and gastritis predispose to the development of cholera [46].

As regards host susceptibility factor, epidemiologic research suggests that there is association between cholera and blood group. Researchers postulated that the incidence of cholera in patients with blood group A was lower than those in the general population, while incidence in those with blood type O was significantly higher. The likelihood of *V. cholera* infection progressing to the severe form, cholera gravis, appears to be related to the individual's ABO blood group. Thus, individuals with blood group O are more likely to exhibit severe diarrhea [47, 48]. No epidemiologic data from studies in Nigeria to suggest an association between individual′s susceptibility to cholera and blood group.

## Management of cholera

The mainstay of the case management of cholera is treatment of dehydration using Oral Rehydration Therapy (ORS) or IV fluids (Ringer lactate) and electrolytes [49]. Although, Oral Rehydration Therapy (ORS) has the advantage of being low-cost and simple, using the technique of decision analysis, Babaniyi concluded that community-based control of Diarrhoeal Diseases interventions in Nigeria is problematic to evaluate [50].

In cholera management, antibiotic prophylaxis is usually not part of intervention but essential for disease treatment in severe cases. However, *Vibrio cholera* strains from endemic and outbreaks situation within the last decade revealed interesting patterns of antibiotic resistance to commonly used antimicrobial agents. Mobile genetic elements able to transfer multiple drug resistance among *Vibrio cholera* strains have also been described in numerous studies and are considered a major public health problem [51]. Eighty six strains of *Vibrio cholera* O1 (79 Ogawa serotype and 7 Inaba serotype) from 1992 outbreak in Nigeria were less sensitive to ampicillin, penicillin, cloxacillin, cotrimoxazole, streptomycin, and tetracycline [52]. The 1995 study also described *V. cholera* strains with 4.5 kilobase to 150 kilobase plasmids specifying resistance to ampicillin, tetracycline, and trimethoprim. Ten of the forty-one isolates were able to transfer resistant plasmids to *Escherichia coli* K-12 by conjugation suggesting that conditions conducive for transmission of resistant strains exist in Nigeria [52].

Obvious emergence of resistant strains could be correlated with widespread therapeutic and prophylactic administration of antibiotics especially tetracycline and their availability over the counter. While continued misuse of antibiotics has indubitably contributed to endemicity most infections, the recent cholera outbreak experience proposes that other factors also play a role in determining whether a particular strain (or resistance plasmid) remains in a given geographic area. Studies are however needed in this area to elucidate this concept.

### Prevention and control measures

In Nigeria, existing prevention and control strategies are multi-sectoral. Epidemic Preparedness and Response (EPR) approaches including registration of cases, case management and public health measures targeting personal hygiene and water treatment as well as emergency responses from both governmental and non-government agencies have contributed to the reduction in case fatality rates over the years and should be sustained. Nevertheless, the need to explore more viable approaches cannot be overplayed if the infection has to be wholly curtailed.

Various studies elsewhere have utilised geographic and mathematical information systems to assess spatial distribution of cholera at local levels, demonstrating case clustering and disease risk areas [53, 54]. Modeling techniques using climate data, remote monitoring, and geographic information systems also provide new techniques that may contribute to the prediction of cholera epidemics [55]. We propose that such models can aid understanding of epidemic processes and help design effective control strategies. Due to its endemicity in Nigeria, surveillance systems can provide early alerts to outbreaks, therefore leading to coordinated response.

More importantly, it is necessary to introduce intervention measures that address the root problems of poor sanitation and unsafe water supplies in order to prevent future cholera epidemics. In this regards, perhaps, prevention of the disease is the best way to counter subsequent outbreaks. Simple measures as boiling the water for drinking, washing and cooking purposes, treatment of infected facilities, sewages and drainage systems, proper disposal of infected materials such as waste products, clothing, and beddings, treatment of infected faecal waste water produced by cholera victims and sterilisation of utensils either by boiling or by using chlorine bleach. Studies have also indicated that use of soap and hand washing promotion can achieve a 26 to 62% decrease in the incidence of diarrhoea in developing countries [56–58].

Understanding the seasonality and location of outbreaks may also provide guidance for improving cholera control activities for vulnerable areas. Vigorous health promotion activities in terms of continuous public enlightenment on cholera are evidently essential to controlling the infection. Health systems need to be strengthened with the provision of adequate manpower, equipment, drugs and consumables. There should also be an improvement on surveillance systems, communication and transport. Mechanisms for quick intervention should be put in place.

Defining strain diversity using molecular biology techniques may also assist with describing the intrinsic characteristics of diseases, such as the persistence of infection. The knowledge of interaction of strain variants may also be critical option for controlling the infection since molecular biology techniques are accessible. This can improve prevention plans, as well as risk assessment for potential cholera outbreaks.

Above all, World Health Organization (WHO) recommends that immunisation with currently available cholera vaccines be used in conjunction with the usually recommended control measures in areas where cholera is endemic as well as in areas at risk of outbreaks [59]. Oral vaccine has been shown to provide short-term protection of 85-90% against *V. cholera* O1 among all age groups at 4-6 months following immunisation with minimal side effects [60].

## Conclusion

Although limited epidemiologic information and studies exist regarding the extent of infection and characteristics of circulating strains in Nigeria, there is clearly a link to poverty, dirty environment, and lack of social amenities including provision of good water sources. These factors definitely imparted much on the frequency and severity of the disease as well as its epidemic potential. In addition, it appears that the clonality of the circulating strains has greater implications on infection control and management of the disease. This is an important area that needs to be addressed and since there are no epidemiologic information of population structure of circulating strain, it therefore seems, at least for now, that cholera will maintain its challenging transmission role. However, we suggest that epidemiological studies should be directed at collecting detailed information on the sources of infection and transmission mode. Public health education should be strengthened and authorities in disease control should establish a channel for communication and reporting. Contact tracing strategies should also be introduced. Lastly, National laboratory should establish data bank for native strains to facilitate comparative analysis with strains from other countries.
